# The Role of the Global Health Development/Eastern Mediterranean Public Health Network and the Eastern Mediterranean Field Epidemiology Training Programs in Preparedness for COVID-19

**DOI:** 10.2196/18503

**Published:** 2020-03-27

**Authors:** Mohannad Al Nsour, Haitham Bashier, Abulwahed Al Serouri, Elfatih Malik, Yousef Khader, Khwaja Saeed, Aamer Ikram, Abdalla Mohammed Abdalla, Abdelmounim Belalia, Bouchra Assarag, Mirza Amir Baig, Sami Almudarra, Kamal Arqoub, Shahd Osman, Ilham Abu-Khader, Dana Shalabi, Yasir Majeed

**Affiliations:** 1 Global Health Development/Eastern Mediterranean Public Health Network Amman Jordan; 2 Yemen Field Epidemiology Training Program Sana’a Yemen; 3 University of Khartoum Khartoum Sudan; 4 Jordan University of Science and Technology Irbid Jordan; 5 Afghanistan Field Epidemiology Training Program Kabul Afghanistan; 6 National Institute of Health Islamabad Pakistan; 7 Federal Ministry of Health Khartoum Sudan; 8 National School of Public Health Rabat Morocco; 9 Pakistan Field Epidemiology and Laboratory Training Program Islamabad Pakistan; 10 Saudi Field Epidemiology Training Program Riyadh Saudi Arabia; 11 Jordan Field Epidemiology Training Program Amman Jordan; 12 Sudan Field Epidemiology Training Program Khartoum Sudan; 13 Eastern Mediterranean Public Health Network Amman Jordan; 14 Iraq Ministry of Health Baghdad Iraq

**Keywords:** COVID-19, outbreak, preparedness, response, public health

## Abstract

The World Health Organization (WHO) declared the current COVID-19 a public health emergency of international concern on January 30, 2020. Countries in the Eastern Mediterranean Region (EMR) have a high vulnerability and variable capacity to respond to outbreaks. Many of these countries addressed the need for increasing capacity in the areas of surveillance and rapid response to public health threats. Moreover, countries addressed the need for communication strategies that direct the public to actions for self- and community protection. This viewpoint article aims to highlight the contribution of the Global Health Development (GHD)/Eastern Mediterranean Public Health Network (EMPHNET) and the EMR’s Field Epidemiology Training Program (FETPs) to prepare for and respond to the current COVID-19 threat. GHD/EMPHNET has the scientific expertise to contribute to elevating the level of country alert and preparedness in the EMR and to provide technical support through health promotion, training and training materials, guidelines, coordination, and communication. The FETPs are currently actively participating in surveillance and screening at the ports of entry, development of communication materials and guidelines, and sharing information to health professionals and the public. However, some countries remain ill-equipped, have poor diagnostic capacity, and are in need of further capacity development in response to public health threats. It is essential that GHD/EMPHNET and FETPs continue building the capacity to respond to COVID-19 and intensify support for preparedness and response to public health emergencies.

## Introduction

### Background

The World Health Organization (WHO) declared the current COVID-19 a public health emergency of international concern on January 30, 2020 [1]. As of March 19, 2020, a total of 220,351 confirmed cases and 8987 deaths were reported [2]. Coronaviruses are a large family of respiratory viruses that can cause diseases such as the Middle East Respiratory Syndrome (MERS) and the Severe Acute Respiratory Syndrome (SARS) [3,4]. The causative agent of the current outbreak, which has originated in Wuhan City in China, was identified as a novel coronavirus on January 7, 2020 [5], and the disease has been named COVID-19.

Many countries worldwide are making every effort to prevent the spread of COVID-19. Several reports of clusters of cases among families and infection of health care workers pointed to the human-to-human transmission of the virus. Infected patients presented mostly with fever, cough, and dyspnea within 7-14 days of exposure to the infection, and few showed upper gastrointestinal symptoms [6]. About 25% of the infected patients, especially the elderly and those with comorbidities, needed intensive care support for acute respiratory symptoms, multiorgan failure, or coinfections [7].

The case fatality rate in the first published report of 99 cases from Wuhan was 11% [8]. Another study reported a mortality rate of 4.3% [9]. It was also estimated that more than 2 new cases are generated by a single infected patient [10]. The probable transmission from individuals before the onset of symptoms or very early minimal symptoms makes COVID-19 much more difficult to control. It reduces the impact of temperature screening and highlights the critical need for accurate contact tracing starting from the day before the onset of symptoms as well as strict quarantine measures and monitoring before more chains of contagion are established.

Because of the exponential increase in the number of cases and deaths, many countries have adopted pandemic preparedness activities and proactive approaches, such as entry restrictions from affected countries; temperature screening at land, air, and sea checkpoints; mandatory leave of absence for travelers within 14 days of their return from affected countries; quarantine of contacts or those deemed to be in the incubation period; and public education and awareness.

A recent study showed that many countries in Africa including some countries that are part of the Eastern Mediterranean Region (EMR) have variable capacity to respond to outbreaks and high vulnerability [11]. Beside the protracted conflicts in many countries in the region, lack of infrastructure, limited resources, inadequate prevention control practices, poor preparedness capacity, and inadequate laboratory infrastructures and resources in many countries in the EMR are among the main barriers to adequately detect and respond to COVID-19. Many of the EMR countries addressed the need for increasing capacity in the areas of surveillance and rapid identification of suspected cases, patient transfer and isolation, rapid diagnosis, tracing and follow-up of potential contacts, strict health facility infection prevention and control, and other active public health control interventions. Moreover, countries addressed the need for communication strategies that provide general populations and vulnerable populations with actionable information for self-protection, including identification of symptoms, and clear guidance for seeking treatment.

### Objectives

This viewpoint aimed to highlight the contribution of the Global Health Development (GHD)/Eastern Mediterranean Public Health Network (EMPHNET) and the EMR’s Field Epidemiology Training Programs (FETPs) to the preparedness capacities in countries in the EMR to respond to the current COVID-19 threat.

## The Role of the GHD/EMPHNET in Preparedness and Response to COVID-19

### Overview

GHD/EMPHNET has been playing an active role in supporting the FETPs across the EMR in their efforts to combat COVID-19 threats. On a technical level, EMPHNET’s Public Health Emergency Management Center (PHEMC) has been the hub for collecting technical information from the programs, disseminating relevant information, and coordinating response efforts. The center also directed attention to new publications and guidelines issued by the WHO and Centers for Disease Control and Prevention (CDC).

GHD/EMPHNET is escalating efforts and activities to support countries in the EMR in preparedness and response to COVID-19 outbreak, through its countries’ FETP graduates and residents. GHD/EMPHNET has the scientific expertise to contribute to elevating the level of country alert and preparedness in the EMR and to provide technical support through promotional material (leaflets and brochures), workshops on contact tracing, dissemination of guidelines, regular sharing of technical updates, development of teaching case-studies to educate public health professionals on COVID-19, and rapid response team training.

A core team was formed to discuss the current situation and updates on COVID-19 emergency on a daily basis and subsequent steps in its response plan. The group comprises the PHEMC and Center of Excellence for Applied Epidemiology alongside representatives from supporting domains like the Knowledge Exchange and Networking. In the regular weekly teleconference communications with FETP’s directors from 10 countries in the EMR, the team discusses updates and any other response activities, discusses and exchanges information, explores and shares the latest technical tools and guidelines with the FETPs, coordinates response efforts at the national and regional levels, and explores additional support and collaboration with partners regarding COVID-19 activities. This team coordinates with the rest of the organization to ensure that efforts made are in their place and of relevance. All these activities are just the first steps taken by the GHD/EMPHNET, FETPs, and other stakeholders to alleviate the effects of coronavirus in the region.

Considering that sharing of relevant knowledge is key in such instances, GHD/EMPHNET has activated its networking platform EpiShares [12] to its full capacity for this cause. Not only has it created a page titled “COVID-19 Updates” to post hourly updates on the virus and its spread, but it has also created a private group titled “FETP Professionals,” which serves as a space for FETP directors, advisors, and coordinators to discuss key issues of concern in this regard. The group also allows its members to upload documents, and it is a space for sharing meeting minutes, meeting agendas, and activities planned in response to the outbreak. Although the group is exclusive to specific members, the page is open for public viewing.

In the area of knowledge sharing, the GHD/EMPHNET also produces daily news round-up that it disseminates widely. The purpose of this update is to provide authentic news and ensure that it is filtered from the rumors that are provided by crowdsourced news platforms. Believing in the significance of FETPs and their work in such instances, GHD/EMPHNET is also publishing weekly bulletins to highlight their achievements.

### The Efforts of FETPs to Respond to COVID-19

The purpose of the FETPs is to increase the epidemiologic capacity of a country’s public health workforce in order to detect and respond to health threats and develop internal expertise in area of field epidemiology [13]. As service-based training programs implement competency-based training under the supervision of qualified mentors/supervisors, these programs focus on the practice of epidemiology in real time and real place. These programs are focused on building workforce capacity to contribute to strengthening their country’s health system to detect, notify, report, and respond to events that threaten the national and international health. They focus on public health surveillance, outbreak investigations, epidemiological methods, laboratory and biosafety, risk communications, health-related surveys, and evaluation of the impact of prevention and control programs. The FETPs’ curricula aim to improve public health systems and develop professional skills to ensure the country meets the surveillance and response requirements. The programs are established within the Ministries of Health and access technical assistance from the CDC.

They play an instrumental role in responding to the current emergency. Being embedded within the ministries of health, national public health institutes, and other public health agencies, the FETPS in Afghanistan, Bangladesh, Egypt, Iraq, Jordan, Morocco, Pakistan, Saudi Arabia, Sudan, Tunisia, and Yemen have been deeply involved in actions responding to COVID-19, including case investigations, points of entry/arrivals screening, isolation protocols, transferring cases, risk communication, and training on infection control.

### Management Functions

For years, FETP graduates have been leading key positions in the public health system in the Ministry of Health at central, governorate, and district levels. During the current event, FETPs in many countries are members of technical committees in Ministries of Health, coordination platforms with various stakeholders, and advisory/higher committees. This enabled the FETPs to directly contribute to the national efforts in managing the COVID-19 threat.

FETPs are directly involved in developing preparedness plans using different scenarios for preparedness and response. In addition, FETP residents and graduates in the region have assisted in developing/adapting local guidelines, protocols, and case definitions for health professionals to implement with various interventions against COVID-19. They have directly assisted in assessing the needs in health facilities and for isolation rooms as well as the preparedness activities, and evaluating the surveillance system to identify the gaps and needs.

### Surveillance

All FETPs in the region play crucial roles in supporting the surveillance functions in their countries at this time of COVID-19 pandemic. Their support is documented at the central, provincial, and even local levels. They are actively engaged in setting up and running the event and case-based surveillance. Moreover, they are engaged in searching for rumors that appear on social media and communicate them to the concerned bodies in the Ministry of Health.

FETP fellows are involved in close monitoring of the global trends of COVID-19 and mortality through relevant websites, with daily monitoring of results of surveillance systems and entry point reports for identifying and following up on suspected cases.

FETPs advisors, graduates, and residents are involved in the management of surveillance data, data analysis, reporting of cases, and development and distribution of a standard case definition for the COVID-19.

The FETP fellows in many countries have been working with hospitals to develop isolation and infection control protocols. They assisted in collecting samples and sent them for laboratory testing to confirm suspected COVID-19 cases.

### Screening and Isolation Centers

FETPs are participating in screening passengers at different points of entries. Their roles range from running the thermal scanners installed at entry points, interviewing arrivals, filling-in surveillance forms, and contacting the arrivals in the follow-up period. FETPs have a direct role in developing and conducting training programs for all those who work at the points of entries, including health workers and workers from other sectors. In addition, FETP fellows participate in training of health workers and organizing and managing the isolation of suspected cases and quarantines for confirmed cases.

### Health Promotion and Education

FETPs have significantly contributed to the design and development of health education messages and promotional materials. FETPs are supporting the direct communication and follow-up with arrivals from abroad and providing them with the needed health messages. In some countries, FETP residents and graduates are responding to public queries through the specified hotlines. FETP residents are also playing a leading role in developing “Question & Answers” documents with standard appropriate information to be used by hotline personnel in response to the expected public’s questions as well as the development of communication materials including brochures and posters.

In some countries, FETPs conducted a series of specialized orientation sessions for health professionals to standardize the protocols, agree on the case definition, and unify the message to the public.

### Research

All residents of the advanced and intermediate levels were engaged in searching for published scientific literature, standard operating procedures, and guidelines, and supported development of the national guidelines for the COVID-19 epidemic. As one of the core competencies, FETPs have started working on different operational research and documents. These research topics are to study the system readiness, knowledge, attitudes, and practices of the health workforce with regard to COVID-19, epidemiology of the disease at the national level, best practices at the points of entries and isolation centers, and infection-control measures.

### Training

FETPs in the entire region are directly and significantly involved in developing training materials and conducting training events for various health professionals. These trainings cover rapid response teams, points of entries, contact tracing, lab and sample management, infection control, cases management, and other processes. The modalities range from in-site training to simulation exercises to practice the countries’ readiness to face the threat of COVID-19.

## Conclusions

The FETPs in the EMR have implemented and are currently working on many activities to strengthen countries’ preparedness against COVID-19. The FETPs participated actively in airport surveillance; implemented temperature screening at ports of entry; developed communication materials and guidelines; and shared information to health professionals and the public, often with a 24-hour dedicated hotlines. However, some countries remain ill-equipped, have poor diagnostic capacity, and are in need of further capacity development in response to public health threats. It is essential that GHD/EMPHNET and FETPS continue building the capacity to respond to COVID-19 and intensify support for preparedness and response to public health emergencies.

## Abbreviations

CDC: Centers for Disease Control and Prevention
EMPHNET: Eastern Mediterranean Public Health Network
EMR: Eastern Mediterranean Region
FETP: Field Epidemiology Training Program
GHD: Global Health Development
MERS: Middle East Respiratory Syndrome
PHEMC: Public Health Emergency Management Center
SARS: Severe Acute Respiratory Syndrome
WHO: World Health Organization
